# Identification of Serum Antibodies Cross-Reactive with Pathogenic Betacoronavirus Amongst Rural Communities Within the Forested Region of the Republic of Guinea

**DOI:** 10.3390/v18090989

**Published:** 2026-09-08

**Authors:** Tom R. W. Tipton, Joseph A. Bore, Joseph Timothy, Stephanie Longet, Stephen M. Laidlaw, Grace Hood, Craig Thompson, Jack Mellors, Ifono Kekoura, Millimon S. Lucien, Beatrice K. Koivogui, Kpade Zeze, David Matthews, Andrew D. Davidson, Alex Mentzer, Donal Skelly, Paul Klenerman, Julian Hiscox, N’Faly Magassouba, Kimberley Fornace, Miles W. Carroll

**Affiliations:** 1 Center for Human Genetics, Nuffield Department of Medicine, University of Oxford, Oxford OX3 7BN, UK; stephanie.longet@univ-st-etienne.fr (S.L.); stephen.laidlaw@well.ox.ac.uk (S.M.L.); grace.hood@well.ox.ac.uk (G.H.); donal.skelly@ndcn.ox.ac.uk (D.S.);; 2 Nuffield Department of Medicine, Pandemic Sciences Institute, University of Oxford, Oxford OX3 7BN, UK; 3 Centre de Recherche et d’Analyse Médicale (CRAM), Macenta, Guinea; jabore34@gmail.com (J.A.B.);; 4 Department of Disease Control, London School of Hygiene and Tropical Medicine, Keppel St, Bloomsbury, London WC1E 7HT, UK; joseph.timothy@lshtm.ac.uk; 5 Warwick Medical School, University of Warwick, Coventry CV4 7AL, UK; 6 School of Biochemistry and Cellular and Molecular Medicine, University of Bristol, Bristol BS8 1TD, UK; d.a.matthews@bristol.ac.uk (D.M.); andrew.davidson@bristol.ac.uk (A.D.D.); 7 Peter Medawar Building for Pathogen Research, Nuffield Department of Medicine, University of Oxford, Oxford OX1 3SY, UK; 8 Institute of Infection, Veterinary & Ecological Sciences, Faculty of Health and Life Sciences, University of Liverpool, Liverpool CH64 7TE, UK; 9 Projet Laboratoire Fièvres Hémorragiques, Conakry, Guinea; 10 Saw Swee Hock School of Public Health, National University of Singapore, Singapore 117549, Singapore; kfornace@nus.edu.sg

**Keywords:** zoonosis, sero-epidemiology, coronavirus

## Abstract

**Objectives**: The SARS-CoV-2 pandemic caused major morbidity, mortality, and economic disruption, highlighting the need to better understand zoonotic spillover risks posed by coronaviruses. We investigated whether archived human sera from forested Guinea contained evidence of prior exposure to coronaviruses antigenically related to known pathogenic human coronaviruses. **Methods**: Archived sera collected in 2017–2018 from wildlife hunter communities in Guinea were analysed using serological assays against spike glycoproteins from SARS-CoV, SARS-CoV-2, MERS-CoV, and seasonal human coronaviruses. Binding responses to receptor binding domains were also assessed, together with spatial and subgroup analyses. **Results**: Pre-pandemic sera showed IgG cross-reactivity to SARS-CoV, SARS-CoV-2, and MERS-CoV spike antigens. A distinct subgroup demonstrated strong binding to both SARS-CoV and MERS-CoV receptor binding domains. **Conclusions**: These findings highlight the utility of sero-epidemiology in identifying potential zoonotic spillover. However, further investigation is needed to identify these viruses and determine their homology to known pathogenic coronaviruses.

## 1. Introduction

Coronaviruses (CoVs) are enveloped positive-sense single-stranded RNA viruses from the family *Coronaviridae* and subfamily *Orthocoronavirinae*. This subfamily contains four genera: *Alpha*-, *Beta*-, *Gamma*- and *Deltacoronaviruses*. Among *Alpha*- and *Betacoronaviruses*, HCoV-229E, HCoV-NL63, HCoV-OC43 and HCoV-HKU1 are endemic in the human population and can cause mild disease, most commonly resulting in cold-like symptoms, but occasionally severe pneumonia has been reported [1]. These seasonal CoVs utilise either Aminopeptidase N (APN/CD13), 9-O-acetylated sialic acid residues or Angiotensin-converting enzyme 2 (ACE2) to mediate cell entry. Importantly, there are three further CoVs known to infect humans: *Severe acute respiratory syndrome coronavirus* (SARS-CoV), *Middle East respiratory syndrome-related coronavirus* (MERS-CoV) and *Severe acute respiratory syndrome coronavirus-2* (SARS-CoV-2). These all belong to the *Betacoronavirus* genus and can often result in severe respiratory disease. SARS-CoV and MERS-CoV were responsible for outbreaks in 2002 and 2012, respectively, whereas the SARS-CoV-2 pandemic began in December 2019 and was declared as a Public Health Emergency of International Concern (PHEIC) in 2023. These CoVs share basic genomic and biologic properties, including structural similarities in their spike protein, which is the primary viral determinant of host cell attachment and entry, though the two SARS-CoVs each utilise the angiotensin-converting enzyme 2 (ACE2) receptor, whereas MERS-CoV utilises the dipeptidyl peptidase 4 (DPP4) receptor [2].

Bats host several SARS-like viruses (belonging to the *Sarbecovirus* subgenus) and are an accepted reservoir for *Betacoronaviruses* [1]. The first zoonotic cases of SARS-CoV were documented in 2002 with strong phylogenetic links to bats as reservoir hosts and human spillover epidemiologically linked to civet cats [3]. Subsequently, MERS-CoV appeared in humans during 2012 with likely origins in African bats but with camels defined as the intermediate for the human spillover event [4,5]. SARS-CoV-2 was first reported amongst humans in late 2019, and its whole genome has ~96% sequence identity with the bat CoV RaTG13 genome [6]. It has been hypothesised that recombination of a pangolin CoV with bat RaTG13-like virus acted as the potential zoonotic source [7]. However, there is debate as to whether pangolins merely served as an intermediate host rather than providing new genetic material or if there is an as-yet-undiscovered bat CoV lineage [8]. In addition, more recent evidence has suggested that racoon dogs acted as the zoonotic origin of SARS-CoV-2 [9,10]. Since the SARS-CoV outbreak in 2002, several studies have been performed to identify the existence of SARS-like viruses in bats outside of China [11]. For example, in 2007 Muller et al. reported the presence of SARS-CoV spike- and nucleocapsid-specific antibody responses in an historical collection of African bat sera dating back to 1990. Their results showed significant antibody responses (average 12%) in the *Rousettus aegyptiacus* species of fruit bats from the Oriental Province of the Democratic Republic of Congo (DRC) and the Limpopo Province of South Africa. Muller et al. also reported that >10% of *Mops condylurus* insectivore bats were positive for antibodies to SARS-CoV from Limpopo and Mpumalanga Provinces, South Africa [12]. In addition, Quan et al. found evidence for SARS-like CoV in leaf-nosed bats collected in Nigeria [13]. Ithete et al. identified a novel MERS-like CoV in a Neoromicia cf. zuluensis bat [14], and the recent PREDICT country report from Guinea identified 10 different CoVs among 25 bats, including four *Betacoronaviruses*, three known and one not previously documented [15].

One of the fundamental challenges of surveillance for these emerging diseases is the low probability of detecting human infections with novel zoonotic viruses, particularly in rural populations where surveillance and health seeking for illness with mild to moderate self-limiting symptoms is scarce. By detecting long-lived antibody responses, serological surveillance has enormous potential to increase the probability of detecting rare spillover events [16]. Although these methods have widely been used to characterise transmission dynamics of zoonotic pathogens with sparse data [17], interpretation of serological data is notoriously difficult due to heterogeneities in individual immune responses, cross-reactivity, assay cut-off thresholds and uncertainty around the time of exposure and antibody kinetics [18]. These limitations can be further amplified when serological methods are used to identify exposure to unknown pathogens without previous described immune responses. However, statistical modelling approaches, such as latent class analysis, are increasingly used to analyse results from imperfect diagnostic tests, enabling estimation of seroprevalence while accounting for uncertainty in infection status [19]. While emerging disease surveillance has predominantly focused on pathogen genomics or the detection of clinical syndromes, integrating serological screening of multi-pathogen antibody responses with these analysis methods can be used to identify populations with evidence of exposure to previously uncharacterised emerging pathogens.

In 2015, we embarked on a longitudinal study of Ebola virus disease (EVD) survivors working with two cohorts in the Republic of Guinea; Gueckedou (an urban city within the forested region of Guinea) and Coyah (an urban area, within the savannah region). In addition, since 2017, we have been performing sero-epidemiology studies in isolated villages in the heavily forested prefecture of Macenta which is adjacent to Gueckedou prefecture within the forested region of the Republic of Guinea. During 2017–2018, we collected blood samples from 517 individuals, concentrating on wildlife hunters and their close family throughout 40 villages in Macenta prefecture. The forested region of Macenta is rich in both pangolins [20] and fruit bats [21]; therefore, we decided to test samples from the forested region, (hereby referred to as Macenta in comparison to those of a more urban cohort (Gueckedou and Coyah) for antibodies reactive to SARS-CoV, SARS-CoV-2, MERS-CoV and seasonal CoV 229E, NL63, OC43, and HKU1 spike protein. This Macenta dataset has previously been published on in the context of emerging filovirus exposure [22].

## 2. Methods

### 2.1. Participants

Serum samples from rural populations were collected in Macenta prefecture in Guinea located in West Africa. Samples were collected from February to December 2017. Within the Macenta region, 517 participants living in 40 villages were enrolled. The volunteers were wildlife hunters and/or their family members, local health care workers and local workmen (carpentry, masonry, blacksmith and merchant). The average age ranged between 25 and 45 years, and there were 292 men and 225 women. In addition, 94 and 66 plasma samples were collected in 2018 from urban populations in Gueckedou and Coyah, respectively. Samples were collected in the context of a longitudinal EVD survivor study. This study was ethically approved by the board of the UK research ethics council as well as the national research for health ethic committee of Guinea (permit Nº012/CENRS/2017). Volunteers were compensated for their loss of time and any inconveniences experienced during sample collection according to Guinean ethics scales. The cohort of COVID-19 convalescents was composed of 51 adults who developed mild symptoms more than 28–69 days prior to the collection of samples. These samples were positive for SARS-CoV-2 by RT-PCR from an upper respiratory tract swab tested. Our cohort of negative controls for SARS-CoV-2 was composed of 103 sera samples collected from adults in the UK in 2014 and 2018.

### 2.2. Serum Sample Processing

Human peripheral blood samples from Guinea were collected at the nearest available health centre. Blood (approx. 5 mL) was collected into BD vacutainer^®^ blood collection tubes (red cap. REF: 367895, Becton Dickenson, Franklin Lakes, NJ, USA). These tubes contained no anticoagulant, and therefore blood could clot within two hours at room temperature. Samples were centrifuged at 2000× *g* for 10 minutes at room temperature. Serum was collected, aliquoted and stored at −20 °C before shipping on dry ice to the United Kingdom.

### 2.3. MSD Assay

A multiplexed MSD immunoassay (MSD, Rockville, MD, USA) was used to measure the responses to SARS-CoV-2, SARS-CoV, MERS-CoV and seasonal CoVs. A MULTI-SPOT^®^ 96-well, 10 Spot Plate was coated with three SARS-CoV-2 antigens (S, RBD, N), SARS-CoV and MERS-CoV spike trimers, and spike proteins from seasonal CoVs OC43S, HKU1, 229E, and NL63, and bovine serum antigen. Antigens were spotted at 200−400 μg/mL (MSD^®^ Coronavirus Plate 3). Multiplex MSD Assays were performed as per the instructions of the manufacturer. To measure IgG antibodies, 96-well plates were blocked with MSD Blocker A for 30 min. Following washing with washing buffer, our samples were diluted 1:500 in diluent buffer and the reference standard, and internal controls were added to the wells. After 2 h of incubation and a washing step, the detection antibody MSD SULFO-TAG™ Anti-Human IgG Antibody (Meso Scale Diagnostics, Rockville, MD, USA) was added. Following washing, MSD GOLD™ Read Buffer B was added and plates read using a MESO^®^ SECTOR S 600 Reader (Meso Scale Diagnostics, Rockville, MD, USA).

As negative populations were not available for seasonal CoV, we defined cut-off values using finite mixture models.

### 2.4. ELISA

#### 2.4.1. Antigens Used for In-House ELISA

Recombinant SARS-CoV-2 trimer spike S1/S2 (amino acids 1-1280, GenBank: MN MN908947) and the SARS-CoV-2 S1 domain were kindly provided by Prof Gavin Screaton. For the trimeric spike protein, the furin cleavage site was removed, two mutations were introduced to enhance expression, and T4 fibritin stabiliser, TwinStrep Tag and an 8-HisTag were introduced. Recombinant SARS-CoV-2 RBD (319–541) Myc-His was developed and kindly provided by MassBiologics (Boston, MA, USA). SARS S1/S2 His-Tag was produced by Immune Tech (IT-002-001p, New York, NY, USA). SARS S1 His-Tag was produced by Native Antigen Company, Oxford, UK (REC31809-500). SARS RBD His-Tag was produced by Sino Biological (40150-V08B2). MERS S1/S2 His-Tag was produced by Sino Biological, Beijing, China (40069-V08B). MERS S1 camel Fc-Tag was produced by Native Antigen Company (REC31847-100). MERS RBD His-Tag was produced by Sino Biological (40071-V08B1).

#### 2.4.2. In-House Spike S1/S2-, S1- and RBD-Specific IgG ELISA

SARS-CoV-2, SARS-CoV and MERS-CoV spike S1/S2-, S1 domain-, RBD-specific IgG responses were determined by ELISA. High-binding 96-well plates (Nunc Maxisorp, 442404, Thermo Fisher Scientific, Waltham, MA, USA) were coated with 50 µL per well of 2 µg/mL antigens in 1X PBS (Thermo Fisher Scientific, Waltham, MA, USA) or PBS 1X (our negative control for background) and incubated overnight at 4 °C. The ELISA plates were washed five times with wash buffer (1X PBS/0.05% Tween 20 (Sigma-Aldrich, St. Louis, MO, USA), (PBS-T)) and blocked with 200 µL blocker 1% casein in 1X PBS (Thermo Fisher Scientific, Waltham, MA, USA) for 1 h at room temperature. After washing, serum samples were diluted 1/50 in 1% blocker casein, and 50 µL of each dilution was added to the antigen-coated plate and incubated for 2 h at room temperature. Following washing, 50 µL anti-human IgG (γ-chain specific) conjugated to AP (Sigma-Aldrich, St. Louis, MO, USA, A3187) was added to the plate and incubated for 1 h at room temperature. After washing, 1 mg/mL of 4-Nitrophenyl Phosphate solution (Sigma N2765) was prepared in 1X Diethanolamine Buffer (Thermo Fisher Scientific, Waltham, MA, USA, 34064) and 100 µL was added. After development, the absorbance at 405 nm was read using Softmax 7.0., and OD_405nm_ values obtained with the plates coated with PBS were subtracted to OD values of plates coated with antigens for each sample. Then, OD values were transformed into arbitrary units (AUs), assigning our positive control an AU of 1 using Softmax 7.0, and the cut-off was determined as the mean of negatives + 5SD.

#### 2.4.3. ACE2 Neutralisation

The MSD ACE2 inhibition assay (MSD, Rockville, MD, USA) was used to determine the ability of samples to displace ACE2 binding to either SARS-CoV-2 spike or SARS-CoV-2 RBD. A similar protocol to the multiplexed MSD immunoassay previously described was used but following a 1 h incubation with samples, and human ACE2 was added for 1 h. Then, MSD GOLD™ Read Buffer B was added and plates read using a MESO^®^ SECTOR S 600 Reader (Meso Scale Diagnostics, Rockville, MD, USA).

#### 2.4.4. Microneutralisation

Sera were heat-inactivated to 56 °C for 30 min to inactivate the complement. Sera were diluted in MEM containing 1% heat-inactivated FCS, and a volume of wildtype SARS-CoV-2 (Victoria/1/2020) or MERS-CoV was added to each well that had previously been determined to produce 100 to 250 virus foci in the non-neutralised control wells of the plate. Neutralisation was then allowed to take place for 1 h at 37 °C. Virus-susceptible monolayers (Vero/E6 Cells) in 96-well plates were exposed to the serum/virus mixture for a further hour at 37 °C. Virus inocula were removed and replaced with overlay (1% *w*/*v* CMC in MEM containing 4% HI FCS). The plates were transferred to a humidified box and incubated for 22–24 h prior to fixing with formaldehyde. Virus foci were visualised using a SARS-CoV-2 antibody (Jack Tan, University Oxford, UK) specific for the SARS-CoV-2 RBD spike protein and a rabbit HRP conjugate, and infected foci were visualised using TrueBlueTM substrate (Thermo Fisher Scientific, Waltham, MA, USA). Stained foci were counted using an ImmunoSpot^®^ S6 Ultra-V Analyzer (BioSPot module) (Cellular Technology Limited (CTL), Shaker Heights, OH, USA) and the resulting counts analysed using SoftMax Pro v7.0 software (Molecular Devices, LLC, San Jose, CA, USA).

#### 2.4.5. Linear Peptide Microarray

Sample acquisition was performed by PePPerPrint using their PEPperCHIP^®^ Pan-Corona spike Protein Microarray (Heidelberg, Germany), which covers the spike proteins of SARS-CoV-2 (UniProt ID: P0DTC2), SARS-CoV (UniProt ID: P59594), MERS-CoV (UniProt ID: A0A140AYZ5), HCoV-OC43 (UniProt ID: P36334), HCoV-HKU1 (UniProt ID: U3NAI2), HCoV-NL63 (UniProt ID: Q6Q1S2) and HCoV-229E (UniProt ID: P15423) elongated with neutral GSGSGSG linkers at the C- and N-terminus and converted into 15 aa peptides with a peptide–peptide overlap of 13 aa. The PEPperCHIP^®^ Pan-Corona spike Protein Microarray contains 4564 different peptides printed in duplicate (9128 peptide spots) and is framed by additional HA (YPYDVPDYAG, 108 spots) and polio (KEVPALTAVETGAT, 106 spots) control peptides.

Plasma were acquired in duplicate at 1:150 and 1:500, and samples were incubated for 16 h at 4 °C and orbital shaking at 140 rpm followed by staining with Goat anti-human IgG (Fc) DyLight680 (0.1 µg/mL), a 45 min staining at RT. Samples were then acquired using an Innopsys InnoScan 710-IR Microarray Scanner (Agilent Technologies, Inc., Santa Clara, CA, USA); a scanning resolution of 20 µm; and a scanning gain of 50 at low laser power (680 nm, red) and 10 at high laser power (800 nm, green). This was followed by subsequent analysis in Microsoft Excel and PyMOL software v2.5 (Schrödinger, LLC, New York, NY, USA).

#### 2.4.6. Latent Class Analysis

We used BLCA to determine whether individuals clustered into different subgroups based on their diagnostic results to all available serological assays. As previously described, samples were classified as either positive or negative for MERS-CoV, SARS-CoV and SARS-CoV-2 antigens using the mean + 3SD of known negative populations. As no negative controls were available for seasonal CoV, finite mixture models were fit to model the distribution of responses as two Gaussian distributions [23]. We defined cut-off values for each seasonal CoV assay as the mean + 3SD of the modelled negative populations. We then fit Bayesian latent class models using the binary classified response data for all 16 assays. As assays detected different pathogens and antigens and results did not show strong evidence of correlation (calculated using Spearman’s Rho, Figure 3A), we assumed the results of each diagnostic test were independent, conditional on an individual’s probability of being exposed. Within this population, there was an unknown number of subgroups representing possible virus exposures. To determine the number of subgroups, we fit competing BLCA models with 1 to 8 latent classes and selected the best fitting model using Bayesian information criterion. Models were fit in a Bayesian framework using Markov chain Monte Carlo (MCMC) with 10,000 iterations, with 5000 burn-in samples. We used uninformative priors specified as *Uniform(0,1)* with models fit as described by [24].

#### 2.4.7. Spatial Analysis and Individual Risk Factors

To examine whether antibody responses were associated with demographic characteristics or bushmeat hunting, we fit models of individual seropositivity to single SARS-CoV and MERS-CoV RBD antigens using a binomial generalised mixed modelling framework with village included as a random effect. As detailed questionnaire data was only available for Macenta, we limited models to this population. As sampling was non-random, we weighted models by the village populations to reflect the probability of inclusion within the survey. As equal numbers of men and women were included, we did not weight by any other factors. Models were fit in R-INLA (Integrated Nested Laplace Approximation) [25] using weakly informative priors of *Normal(0,10)* for fixed effects. Models were fit in a forward stepwise manner, and final models were selected based on the deviance information criterion (DIC).

Next, to assess spatial patterns of exposure, we fit binomial models to village-level prevalence for each antigen, weighted by village population. We represented the spatial process by Gaussian Markov random fields (GMRFs) using a stochastic partial differential equation (SPDE) approach implemented in INLA. To evaluate evidence of spatial structure in seroprevalence against each antigen, we compared models including a spatial effect with null models and selected models based on DIC. As village-level responses to MERS-CoV and SARS-CoV antigens showed strong evidence of spatial correlation and BLCA identified a subgroup of individuals with elevated responses to these antigens, we developed a hierarchical modelling approach integrating the probability of membership within this subgroup (Group 3 in Supplementary Table S3) within a spatial framework [26]. The final model was fit using MCMC within INLA implemented in R, using 5000 posterior samples to estimate posterior probabilities [27].

## 3. Results

**Figure 1:**
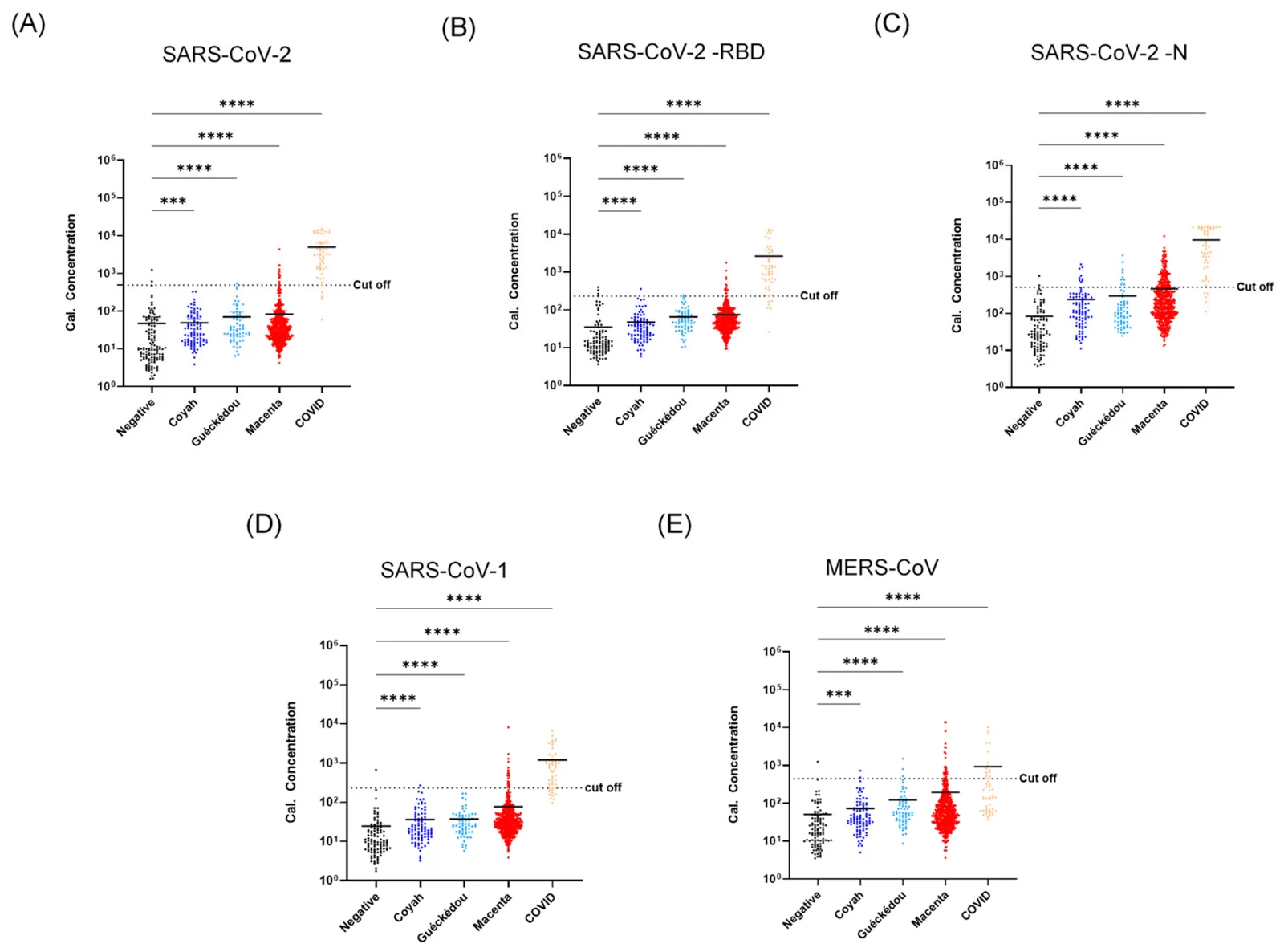
**Total spike and nucleocapsid (N) binding antibodies (IgG) of cohort against various *Beta-coronaviruses.*** Meso Scale Discovery (MSD) analysis was performed on n = 103 UK negative controls (negative), 517 rural forested region samples (Macenta), 66 and 94 samples from the urban areas of Coyah and Gueckedou and 51 UK COVID-19 convalescent samples (COVID-19). The responses to various antigens are displayed as follows: (**A**) SARS-CoV-2 spike; (**B**) SARS-CoV-2 RBD; (**C**) SARS-CoV-2 N; (**D**) SARS-CoV spike; (**E**) MERS-CoV spike. Data are shown as a calculated concentration using a known reference serum and a 4PL fit curve; dashed line represents our in-house cut-off between negative and positive and is determined as the mean of the negative population plus five SD. Mean with standard deviation (SD) is displayed. Statistical analysis used Kruskal–Wallis with multiple comparisons; all groups showed statistical significance vs. the negative group across all antigens tested. *p* = ≤ 0.001 (***) and *p* = ≤ 0.0001 (****).

We set out to test if the Macenta cohort (Table 1) contained serum antibodies (IgG) that were reactive to SARS-CoV, SARS-CoV-2, MERS-CoV or seasonal CoV antigens. To do this we used a multiplex MSD platform to assay samples from Macenta along with samples from Coyah, Gueckadou, SARS-CoV-2 negatives and SARS-CoV-2 convalescents (Figure 1). As expected, COVID-19 convalescent serum samples (collected in the UK, 28–69 days post infection) had detectable IgG responses to the SARS-CoV-2 spike protein, receptor-binding domain (RBD) and nucleocapsid protein (N). This group of COVID-19 convalescent samples also had an elevated response to the SARS-CoV and MERS-CoV spike proteins when compared to negative controls, highlighting the cross-reactive profile of COVID-19 convalescents to similar pathogenic CoV’s. Interestingly, COVID-19 convalescent samples had an elevated response to three out of the four seasonal circulating CoV spike proteins (229E, OC43 and HKU1), again when compared to pre-pandemic, UK healthcare worker negative controls (Supplementary Figure S1). Amongst the Macenta group, a greater percentage of samples show reactivity to SARS-CoV-2 spike and N antigens above our defined cut-off value, which was determined as the mean of the UK negatives plus five standard deviations (Supplementary Table S1). It is also important to note that the rural sample population of Macenta, as well as those in the urban areas of Coyah and Gueckedou, all had elevated responses, when compared to UK negative controls, to the four seasonal CoVs, which were similar to that of COVID-19 convalescent samples (Supplementary Figure S1). However, there was no evidence of a correlation between the SARS-CoV, SARS-CoV-2 and MERS-CoV response vs. seasonal CoV response (Supplementary Figure S2).

**Figure 2:**
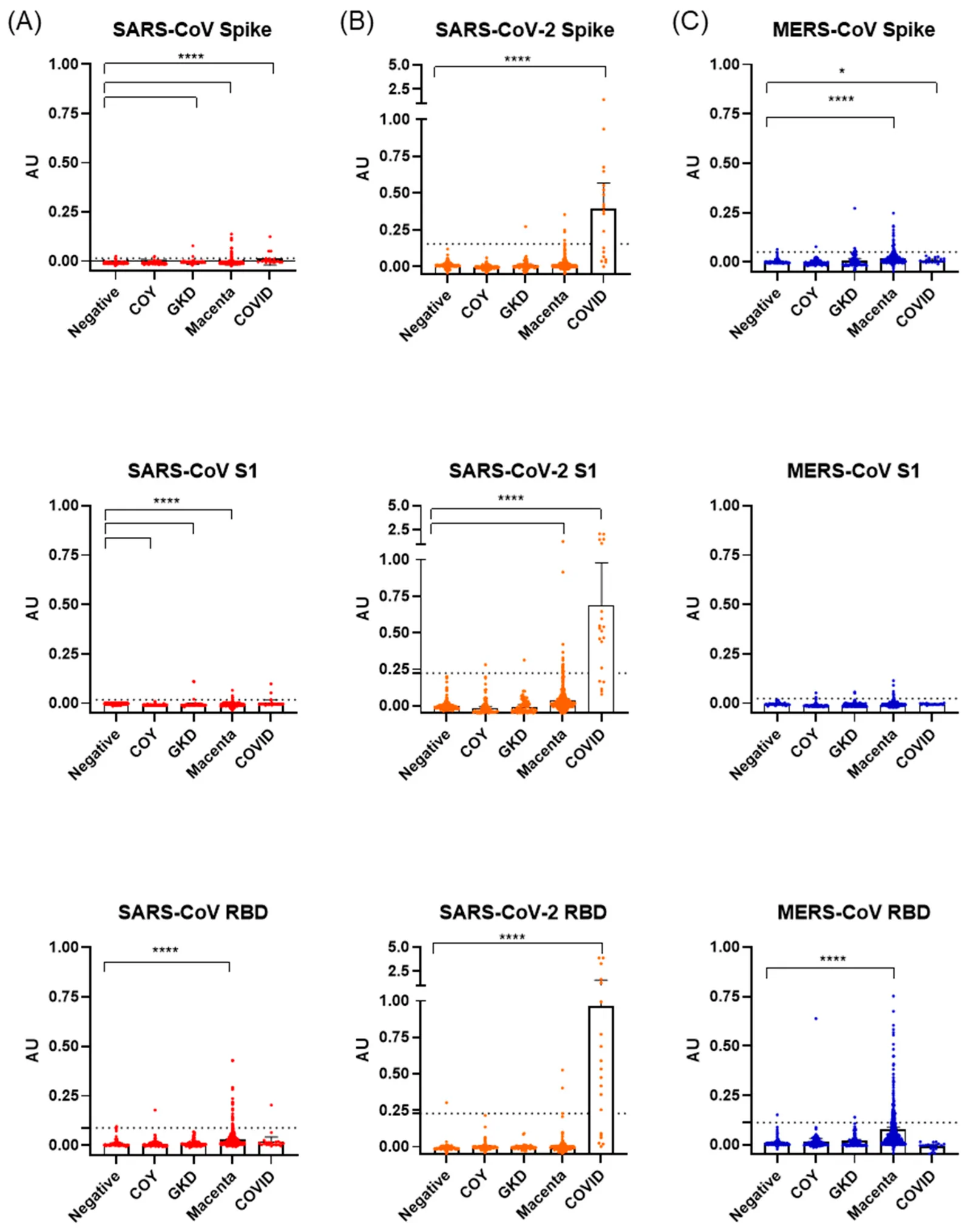
**ELISA analysis of SARS-CoV, SARS-CoV-2 and MERS-CoV spike proteins.** ELISA analysis was performed on 103 UK negative controls, 517 rural forested region samples (Macenta), 66 and 94 samples from the urban areas of Coyah (COY) and Gueckedou (GKD), and 18 COVID-19 convalescent samples (COVID-19). The response to various antigens is displayed as follows: (**A**) SARS-CoV spike, S1 only or RBD; (**B**) SARS-CoV-2 spike, S1 only or RBD; and (**C**) MERS-CoV spike, S1 only or RBD. Values are displayed as arbitrary units: a 3PL fit curve using an in-house standard was used to calculate values, and an AU of 1 would be equivalent activity to the standard. The dashed line represents the in-house cut-off value and is based on the mean of the negatives plus five SD. The mean and 95% CI are displayed. Statistical analysis used Kruskal–Wallis with multiple comparisons; significant differences to the negative group are shown. Where error bars are not present significance did not reach ≤*p* = 0.05. *p* = ≤ 0.05 (*) and *p* = ≤ 0.0001 (****).

The capacity for antibody responses related to common CoV to cross-react with the SARS-CoV-2 spike protein has been documented [28], and it has been found that the majority of cross-reactive antibodies are to the conserved S2 domain with minimal cross-reactive clones to the S1 or RBD regions [29]. To further determine if the Macenta cohort had been exposed to a CoV species related to SARS-CoV, SARS-CoV-2 or MERS-CoV, we performed in-house ELISA to either the spike S1/S2, S1 only or RBD regions of SARS-CoV, SARS-CoV-2 or MERS-CoV (Figure 2, Figure S3). To better account for any background effect, a PBS control was used for each plasma sample to show the plasma background binding to plastic, and this value was subtracted from the antigen bound value. With regard to the response to SARS-CoV antigens, Figure 2 shows that West African samples and UK convalescents have elevated binding to SARS-CoV spike as well as to the SARS-CoV S1 and RBD regions. With regard to SARS-CoV-2 antigens, elevated binding to the spike S1 region was measured amongst Macenta samples compared to the negative controls, and as expected, response amongst COVID-19 convalescents vs. negatives for all SARS-CoV-2 antigens. For the MERS-CoV antigens, there is a difference between Macenta samples and negatives for spike and RBD antigens, but not S1 only, whilst the RBD-specific response appears to be greatly elevated compared to urban Guinea and negative controls. Supplementary Table S2 shows the percentage of positive samples to each of these antigens within each group. Furthermore, amongst the top MERS-CoV spike and RBD binders from these Macenta samples we performed a novel flow cytometer-based bead array, which further confirmed binding to the RBD and activation of the complement system, as measured by C3 deposition [30] (Supplementary Figure S4).

To determine if there was any grouping of antigenically similar responses, we used Bayesian latent class analysis (BLCA) to identify subpopulations of exposed individuals within samples collected from Guinea. BLCA is a method of model-based clustering used to identify homogenous subgroups within populations and is widely applied to estimate infection prevalence from diagnostic tests without gold standards [31]. Rather than classifying exposure status, BLCA uses diagnostic results to calculate probabilities of membership to different unobserved subgroups of the population (latent classes). Using a BLCA approach, we identified four subpopulations using binary results of all serological assays (MSD and in-house ELISA) run on samples from Macenta, Gueckedou and Coyah (Supplementary Table S3). Identified subgroups included a group of 2.3% (SD: 0.804%) of the population with elevated responses to SARS-CoV-2 antigens and an additional group comprising 12.2% (SD: 1.805%) of the population with high responses to MERS-CoV and SARS-CoV antigens. The majority of the population (69.4%, SD 3.612%) had low responses to all CoVs, and 16.0% (SD: 3.311%) had elevated responses only to seasonal CoVs. Individuals with the highest probabilities of membership in subgroups responding to SARS-CoV and MERS-CoV antigens were primarily concentrated in rural Macenta.

**Figure 3:**
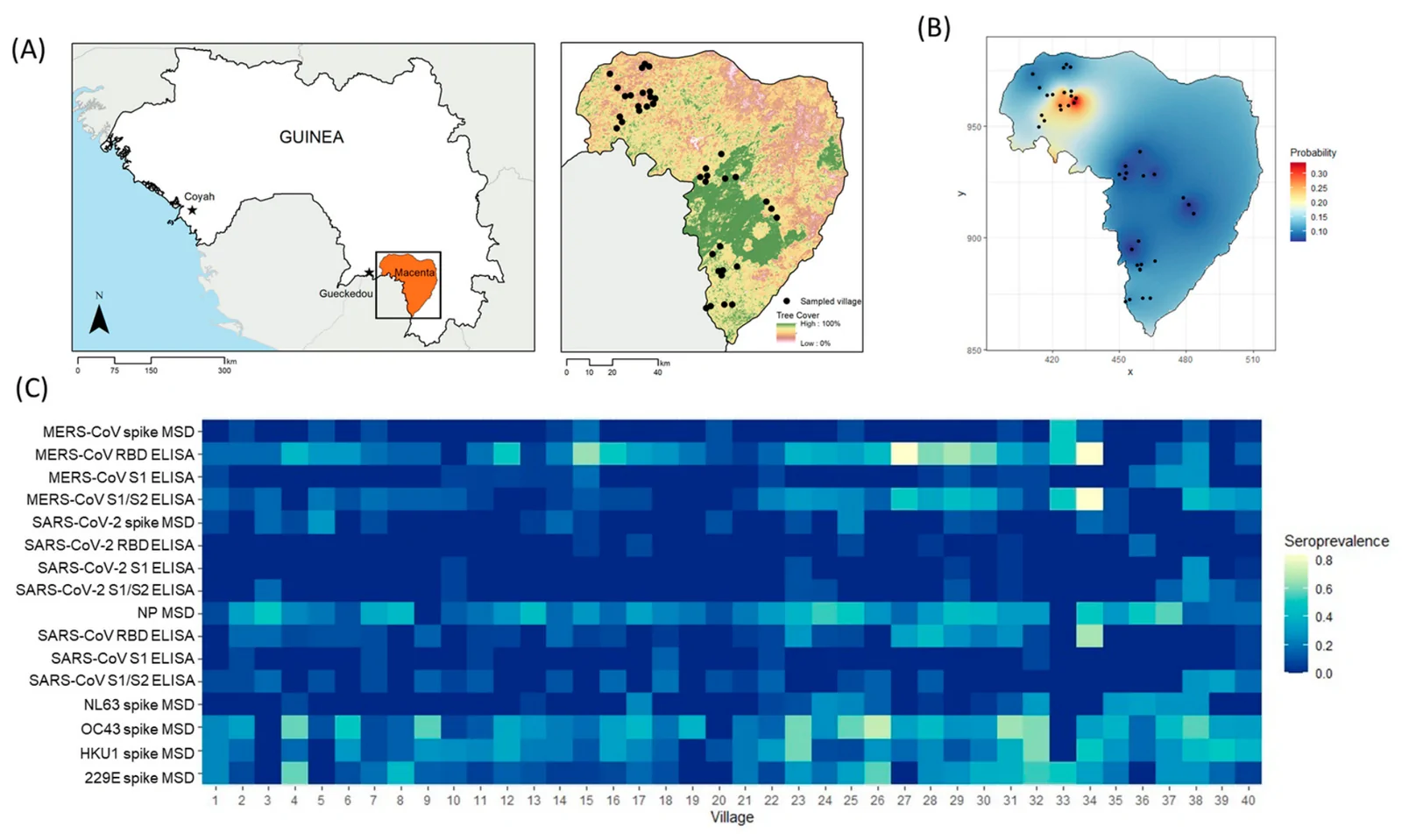
**Correlation and spatial analysis of rural Guinean samples.** (**A**) Geographical map of the republic of Guinea, showing the locations of Coyah and Gueckadou (black star) along with an insert of the Macenta region showing the location of individual villages. (**B**) Mean posterior estimate of probability of individuals belonging to the subgroup with elevated responses to SARS-CoV and MERS-CoV antigens. (**C**) Macenta village-level seroprevalence of responses to all assays used.

To examine the spatial distribution of antibody responses within villages of Macenta (Figure 3A), we first fit hierarchical Bayesian geostatistical models of both single antigen seroprevalence (using binary classifications) before developing models integrating probabilities of membership to the group responding strongly to both SARS-CoV and MERS-CoV antigens. Heterogeneities in antibody responses were detected across all surveyed villages (Figure 3C). From models of single antigen seroprevalence, there was no spatial structure in responses to SARS-CoV-2 antigens or SARS-CoV S1 or MERS-CoV S1 antigens. However, models of seroprevalence based on single antigen assays for other MERS-CoV and SARS-CoV antigens were strongly spatially correlated (as measured by Moran’s I, a statistic that quantifies spatial autocorrelation), with spatially structured random effects improving model fits (Figure 3B). Seasonal CoVs, 229E, HKU1 and OC43 were also spatially correlated, although with distinct distributions from other CoV responses (Supplementary Figure S6). A hierarchical model incorporating the probability of membership to the group responding strongly to SARS-CoV and MERS-CoV RBD antigens identified strong spatial correlation and foci of exposure within Macenta (Figure 3C). Models of individual-level data did not show an association between demographic characteristics or wildlife hunting and probabilities of membership within this exposed group. Despite being located with the forested region, proximity to forest and tree cover were not associated with increased risks.

To determine if these cross-reactive antibody responses found in Macenta samples would have any functional effect against SARS-CoV-2, SARS-CoV and/or MERS-CoV, we performed ACE2-RBD/spike inhibition assays and SARS-CoV-2 or MERS-CoV live virus neutralisation assays using the top SARS-CoV/MERS-CoV reactive samples from the Macenta group, as defined by BLCA analysis (Supplemental Figure S7). We found that, when compared to the COVID-19 convalescent group, the Macenta samples show a significantly decreased ability to inhibit ACE2 binding to either the SARS-CoV-2 spike or RBD protein. In support of this data, we found that samples again fell below our positive detection limit for live virus neutralisation assays, again supporting the inability of these samples to neutralise a live virus.

**Figure 4:**
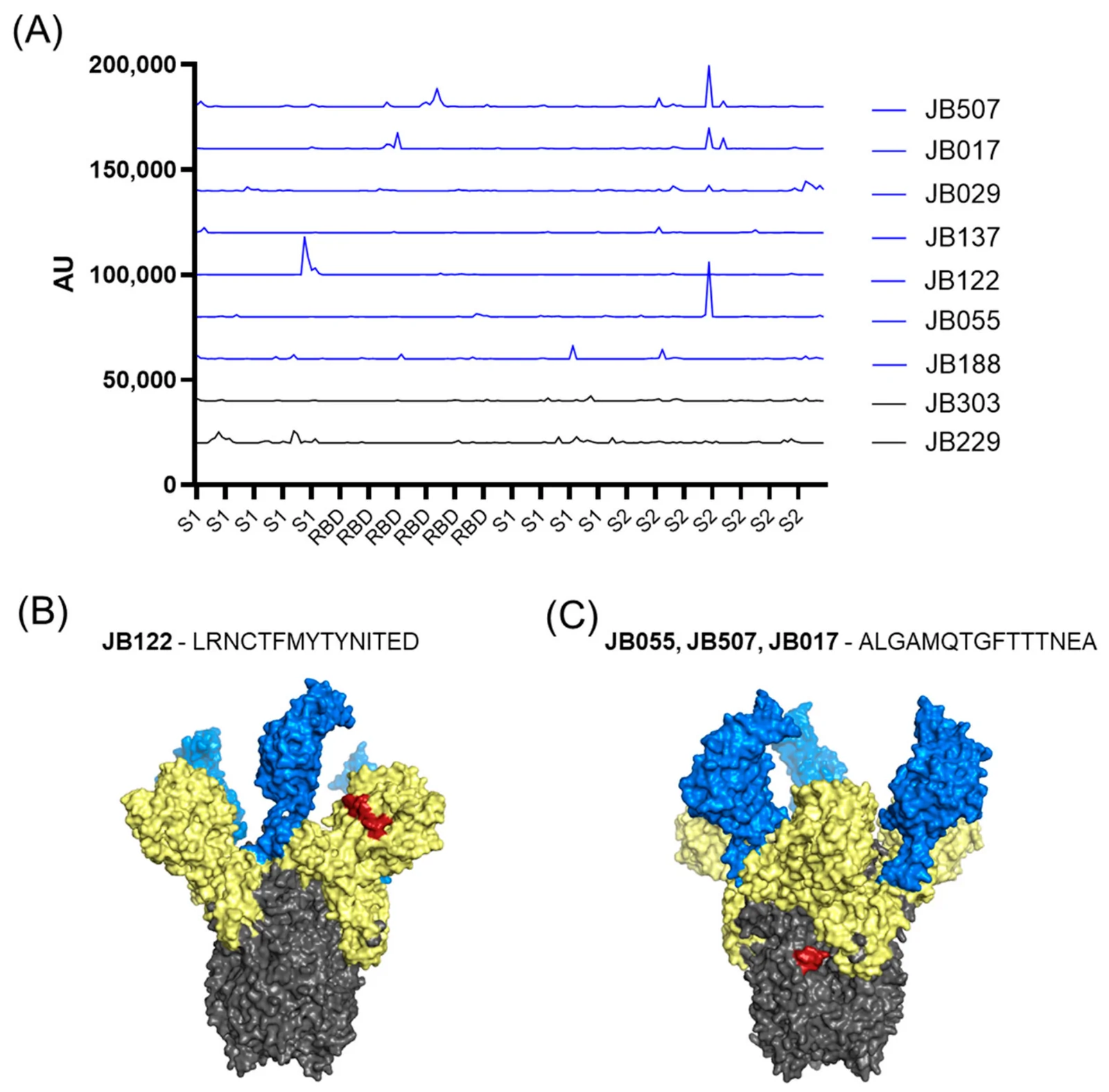
**Mapping antibody binding of MERS-CoV reactive samples using a linear peptide array.** (**A**) We subjected nine samples to a linear peptide array: seven were reactive to either SARS-CoV, SARS-CoV-2 or MERS-CoV glycoprotein (blue) and two were unreactive by ELISA and were used as a negative control (black). Samples were sent for processing at PEPperPRINT and were assayed against a linear peptide array of 15 amino acids off-set by two amino acids and spanning the glycoproteins of SARS-CoV, SARS-CoV-2, MERS-CoV, HKU1, 229E, NL63 and OC43. Following acquisition, sample peptides with a mean sasa of greater than 30% were included in downstream analysis. (**B**,**C**) Mapping of antibody binding regions for JB122 and JB055 using PyMOL v2.5 (yellow = S1, blue = RBD, red = antibody binding site).

Absent neutralisation responses were perhaps unsurprising given the known abrogation in neutralising activity between variants of the same SARS-CoV-2 virus; therefore, in an attempt to further support the hypothesis that we have detected cross-reactive antibodies to a ***Betacoronaviruses***, we sought to identify binding epitopes distinct from seasonal CoVs. To do this, we subjected plasma that bound to MERS-CoV, SARS-CoV or SARS-CoV-2 antigens to a linear peptide microarray (Figure 4, Figure S8). This microarray contained 15 mer peptides off-set by two amino acids and spanning the spike glycoproteins of SARS-CoV, MERS-CoV, SARS-CoV-2, HKU1, NL63, 229E and OC43. From the raw data we excluded any peptides that had a mean solvent assessable area (SASA) of less than 30%; therefore, we can be confident that any remaining peptides are likely to be located at the protein surface and able to interact with antibodies. The results in Figure 4A demonstrate that we were able to identify a number of linear epitopes capable of binding the MERS-CoV spike glycoprotein, and these epitopes (Figure 4B,C) had no homology with the seasonal human CoVs (Supplemental Figure S9).

## 4. Discussion

Together our data show that a high proportion of Macenta samples from 2017 to 2018 bound to SARS-CoV and MERS-CoV spike and RBD with no correlation to seasonal CoV exposure; this may suggest that these antibodies were raised in response to exposure from zoonotic viruses that are more closely related to clade B or C *Betacoronaviruses* such as SARS-CoV or MERS-CoV. However, it is important to note that the absence or a correlation between SARS-CoV, SARS-CoV-2 or MERS-CoV responses and seasonal CoV responses is not direct evidence of this, and further work would be needed to prove these responses are not due to seasonal CoV exposure. We identified clear subpopulations of exposed individuals and demonstrate that responses to these antigens cluster both within individuals and between spatially contiguous villages in the study site. We also note that within Guinea the exposure to seasonal CoVs is similar for both the forested and urban areas and that those sampled in the urban areas had low probabilities of belonging to this exposed subpopulation. Although these data cannot demonstrate transmission routes conclusively, the relatively high overall seroprevalence (~12%) and the markedly higher prevalence in the more urbanised north-western villages suggest that human-to-human transmission may contribute to the observed patterns. This interpretation is supported by the absence of associations with ecological exposures, including hunting, and aligns with the idea that more densely populated settings could facilitate spread. This would be consistent with reports of spillover events of novel viruses leading to stuttering chains of transmission and limited outbreaks [32]. In support of these conclusions is data from Borrega et al., who showed that within a geographically similar cohort there was ~50% cross-reactivity to the N of either SARS-CoV, SARS-CoV-2 or MERS-CoV, as well as detectable neutralisation in a pseudovirus assay [33]. The lack of neutralisation to SARS-CoV-2 or MERS-CoV in this study is perhaps unsurprising due to the low-magnitude responses detected; a possible explanation for this would be that the homology between any regional *Betacoronaviruses* and SARS-CoV-2 or MERS-CoV is not sufficient enough to readily allow any neutralisation, although further sampling and identification of such viruses would be needed to confirm this.

With regard to the circulation of ***Betacoronaviruses*** West Africa, it has been shown that a number of abattoir workers in Nigeria who had exposure to dromedaries showed evidence of an anti-MERS-CoV T cell response [34]. The geographical roots of MERS-CoV are thought to be in Africa, and El-Kafraway et al. phylogenetically mapped distinct strains of MERS-CoV circulating in camel trains as far west as Burkina Faso. None of the putative ancestral MERS-CoV bat reservoirs are found in Guinea, but some (*Taphozius perforates*) are found in Burkina Faso and then as far west as Senegal [35]. Interestingly, a South African MERS-CoV like strain was found in a bat species that is also found in Guinea (Cape serotine) and has significant recombination in the S1 subunit; furthermore, the recent PREDICT report for Guinea identified a number of *Betacoronaviruses* amongst local bats [15]. There is no serological evidence that SARS-CoV-like viruses have been circulating outside those defined regions affected by the SARS-CoV and MERS-CoV outbreaks, and it would be highly unlikely that this rural Guinean population within the forested region has mounted an antibody response following exposure to SARS-CoV, SARS-CoV-2 or MERS-CoV but rather other related ***Betacoronaviruses***. Interestingly, cross-reactivity observed to SARS-CoV and MERS-CoV RBD was not translated to the respective S1 domain. This may be due to the conformation of the S1 domain on the plastic, which may not expose the RBD epitope. Alternatively, since the S1 proteins were produced in mammalian cells and the RBD proteins produced in insect cells (Table 2), protein glycosylation may play a role in these confounding results.

Cross-reactivity between antibodies to different seasonal human CoVs may explain our results and is an important consideration. For example, Che et al. found that SARS-CoV convalescent serum showed cross-reactivity to the N of HCoV-OC43 but not vice versa [36]. Chan et al. demonstrated that 12/20 SARS-CoV patients had antibody titres to HCoV-OC43, 229E or both, but OC43, 229E patient serum did not show cross-reactivity to SARS-CoV [37]. It has also been shown that serum from SARS-CoV convalescent patients can cross-react with that of MERS-CoV [38] and that SARS-CoV serum can cross-react with SARS-CoV-2. However, SARS-CoV-2 serum was shown not to cross-react with the RBD of SARS-CoV or MERS-CoV, suggesting RBD antibody binding [39]. Interestingly, amongst the COVID-19 convalescent samples in our study we can see that there is a cross-reactive response to both SARS-CoV and MERS-CoV spike and that responses to three of the seasonal human CoVs are elevated. To what extent the COVID-19 convalescent baseline antibody levels to these seasonal CoVs were prior to SARS-CoV-2 exposure in this study is unknown, but they are likely to be similar to that of the UK negative controls. As responses to SARS-CoV-2 antigens and SARS-CoV and MERS-CoV RBD antigens showed distinctly different patterns from seasonal CoVs both between individuals and in spatial patterns of prevalence, these may not be solely cross-reactive responses.

The mean IgG response to CoV spike proteins and SARS-CoV-2 N (Figure 1) amongst all Guinean samples is generally higher than that of UK negative samples. Therefore, caution would need to be taken when performing serological studies for SARS-CoV-2 in this region. A similar conclusion was also reached by Ndaye et al. whilst looking at healthcare workers in the DRC [40]. Elevated background antibody binding in West African cohorts has been reported previously and may reflect a combination of increased exposure to diverse pathogens, polyclonal B-cell activation, and immune imprinting from repeated infections. In particular, chronic or repeated Plasmodium infection has been associated with hypergammaglobulinaemia and increased non-specific or cross-reactive antibody binding, including to viral antigens [41]. Malaria-associated immune activation has also been shown to generate antibodies that cross-react with carbohydrate or conserved protein epitopes, potentially enhancing seroreactivity in immunoassays [42]. A limitation of this study is that we use Western negative controls to define the cut-off values in our serological assays, and it may have been more appropriate to use the urban cohort of Coyah to generate assay cut-off values.

Further studies are needed to complement the PREDICT study [15] to determine whether these responses represent exposure to single or multiple pathogens and to estimate wider community-level risks. Given that seropositive cases are clearly not limited to the spatial prevalence hotspots, and that prevalence is ~12% of the main group, then it is reasonable to assume there is/was wider transmission in Macenta and that therefore notable prevalence may be expected at the population level. However, it is important to remember that BLCA identifies statistical groupings based on shared patterns of antibody response and does not provide direct evidence of infection with specific viruses. As with all latent class approaches, the identified groups are model-derived and depend on assumptions regarding the underlying population structure. An important assumption is conditional independence, whereby serological assay results are assumed to be independent once the underlying, unobserved exposure status has been accounted for. This assumption may not always hold because serological assays can exhibit residual correlation due to cross-reactivity or other shared biological and technical factors. Although the four-class model showed the best statistical fit, these unobserved classes should be interpreted as representing patterns of shared serological responses rather than definitive evidence of distinct viral exposures.

We believe this is a novel finding that may have public health implications for the international community. Furthermore, these results also shed light on the distribution of SARS and MERS-like CoVs in addition to the importance of sampling the wildlife in this region of West Africa where spillover events of other high-risk pathogens are known to occur. Additionally, our results highlight the utility of serological surveillance for monitoring patterns of spillover and transmission of unknown viruses and defining future priorities for targeted wildlife and human studies. Therefore, future research should be carried out to identify these potentially novel CoVs and to determine the full extent of serology in these regions.

## Figures and Tables

**Table 1:** Details on the cohorts used in this study.

	Number	Mean Age	Male	Female	Unknown
Negative	103	Not known	Not known	Not known	Not known
Sample	517	39	221	229	7
Coyah	94	Not known	Not known	Not known	Not known
Gueckadou	66	Not known	Not known	Not known	Not known
COVID-19	51	Not known	Not known	Not known	Not known

**Table 2:** Source of antigens used in our in-house ELISA.

Antigen	Accession No.	Cat No	Company/University	Production
SARS-CoV-2 S1/S2	MN MN908947	-	Oxford	HEK293 cells
SARS-CoV-2 S1	MN MN908947	-	Oxford	HEK293 cells
SARS-CoV-2 RBD	MN MN908947	-	Mass Biologics	HEK293 cells
SARS-CoV S1-S2	AY278487	IT-002-001p	Immune Tech	HEK293 cells
SARS-CoV S1	Not listed by manufacturer	REC31809-500	Native Antigen Company	HEK293 cells
SARS-CoV RBD	AAX16192.1	40150-V08B2	Sino Biological	Insect cells
MERS S1/S2	AFS88936.1	40069-V08B	Sino Biological	Insect cells
MERS S1	Not listed by manufacturer	REC31847-100	Native Antigen Company	HEK293 cells
MERS RBD	AFS88936.1	40071-V08B1	Sino Biological	Insect cells

## Data Availability

Data is on FigShare https://doi.org/10.6084/m9.figshare.33477502.

## Supplementary Materials

The following supporting information can be downloaded at https://www.mdpi.com/article/10.3390/v18090989/s1.

## References

[1] Chan J.F., Lau S.K., To K.K., Cheng V.C., Woo P.C., Yuen K.Y. (2015). Middle East respiratory syndrome coronavirus: Another zoonotic betacoronavirus causing SARS-like disease. Clin. Microbiol. Rev..

[2] Butler D. (2013). Receptor for new coronavirus found. Nature.

[3] Wang L.F., Eaton B.T. (2007). Bats, civets and the emergence of SARS. Curr. Top. Microbiol. Immunol..

[4] Goldstein S.A., Weiss S.R. (2017). Origins and pathogenesis of Middle East respiratory syndrome-associated coronavirus: Recent advances. F1000Research.

[5] Corman V.M., Ithete N.L., Richards L.R., Schoeman M.C., Preiser W., Drosten C., Drexler J.F. (2014). Rooting the phylogenetic tree of middle East respiratory syndrome coronavirus by characterization of a conspecific virus from an African bat. J. Virol..

[6] Zhou P., Yang X.L., Wang X.G., Hu B., Zhang L., Zhang W., Si H.R., Zhu Y., Li B., Huang C.L. (2020). A pneumonia outbreak associated with a new coronavirus of probable bat origin. Nature.

[7] Zhang T., Wu Q., Zhang Z. (2020). Probable Pangolin Origin of SARS-CoV-2 Associated with the COVID-19 Outbreak. Curr. Biol..

[8] Boni M.F., Lemey P., Jiang X., Lam T.T., Perry B.W., Castoe T.A., Rambaut A., Robertson D.L. (2020). Evolutionary origins of the SARS-CoV-2 sarbecovirus lineage responsible for the COVID-19 pandemic. Nat. Microbiol..

[9] Pekar J.E., Magee A., Parker E., Moshiri N., Izhikevich K., Havens J.L., Gangavarapu K., Malpica Serrano L.M., Crits-Christoph A., Matteson N.L. (2022). The molecular epidemiology of multiple zoonotic origins of SARS-CoV-2. Science.

[10] Worobey M., Levy J.I., Malpica Serrano L., Crits-Christoph A., Pekar J.E., Goldstein S.A., Rasmussen A.L., Kraemer M.U.G., Newman C., Koopmans M.P.G. (2022). The Huanan Seafood Wholesale Market in Wuhan was the early epicenter of the COVID-19 pandemic. Science.

[11] Tong S., Conrardy C., Ruone S., Kuzmin I.V., Guo X., Tao Y., Niezgoda M., Haynes L., Agwanda B., Breiman R.F. (2009). Detection of novel SARS-like and other coronaviruses in bats from Kenya. Emerg. Infect. Dis..

[12] Muller M.A., Paweska J.T., Leman P.A., Drosten C., Grywna K., Kemp A., Braack L., Sonnenberg K., Niedrig M., Swanepoel R. (2007). Coronavirus antibodies in African bat species. Emerg. Infect. Dis..

[13] Quan P.L., Firth C., Street C., Henriquez J.A., Petrosov A., Tashmukhamedova A., Hutchison S.K., Egholm M., Osinubi M.O., Niezgoda M. (2010). Identification of a severe acute respiratory syndrome coronavirus-like virus in a leaf-nosed bat in Nigeria. mBio.

[14] Ithete N.L., Stoffberg S., Corman V.M., Cottontail V.M., Richards L.R., Schoeman M.C., Drosten C., Drexler J.F., Preiser W. (2013). Close relative of human Middle East respiratory syndrome coronavirus in bat, South Africa. Emerg. Infect. Dis..

[15] USAID Predict Guinea: One Health in Action (2016–20). https://ohi.vetmed.ucdavis.edu/sites/g/files/dgvnsk5251/files/inline-files/PREDICT%20LEGACY%20-%20FINAL%20FOR%20WEB%20-compressed_0.pdf?utm_source=chatgpt.com.

[16] Metcalf C.J., Farrar J., Cutts F.T., Basta N.E., Graham A.L., Lessler J., Ferguson N.M., Burke D.S., Grenfell B.T. (2016). Use of serological surveys to generate key insights into the changing global landscape of infectious disease. Lancet.

[17] Pepin K.M., Kay S.L., Golas B.D., Shriner S.S., Gilbert A.T., Miller R.S., Graham A.L., Riley S., Cross P.C., Samuel M.D. (2017). Inferring infection hazard in wildlife populations by linking data across individual and population scales. Ecol. Lett..

[18] Gilbert A.T., Fooks A.R., Hayman D.T., Horton D.L., Muller T., Plowright R., Peel A.J., Bowen R., Wood J.L., Mills J. (2013). Deciphering serology to understand the ecology of infectious diseases in wildlife. Ecohealth.

[19] Viana M., Mancy R., Biek R., Cleaveland S., Cross P.C., Lloyd-Smith J.O., Haydon D.T. (2014). Assembling evidence for identifying reservoirs of infection. Trends Ecol. Evol..

[20] Ingram D., Cronin D., Challender D., Venditti D., Gonder M. (2019). Characterising trafficking and trade of pangolins in the Gulf of Guinea. Glob. Ecol. Conserv..

[21] Decher J., Hoffmann A., Schaer J., Norris R.W., Kadjo B., Astrin J., Monadjem A., Hutterer R. (2015). Bat Diversity in the Simandou Mountain Range of Guinea, with the Description of a New White-Winged Vespertilionid. Acta Chiropterologica.

[22] Akoi Bore J., Timothy J.W.S., Tipton T., Kekoura I., Hall Y., Hood G., Longet S., Fornace K., Lucien M.S., Fehling S.K. (2024). Serological evidence of zoonotic filovirus exposure among bushmeat hunters in Guinea. Nat. Commun..

[23] Sepulveda N., Stresman G., White M.T., Drakeley C.J. (2015). Current Mathematical Models for Analyzing Anti-Malarial Antibody Data with an Eye to Malaria Elimination and Eradication. J. Immunol. Res..

[24] Li Y., Lord-Bessen J., Shiyko M., Loeb R. (2018). Bayesian Latent Class Analysis Tutorial. Multivar. Behav. Res..

[25] Rue H., Martino S., Chopin N. (2009). Approximate Bayesian inference for latent Gaussian models by using integrated nested Laplace approximations. J. R. Stat. Soc. Ser. B.

[26] Wall M.M., Liu X. (2009). Spatial Latent Class Analysis Model for Spatially Distributed Multivariate Binary Data. Comput. Stat. Data Anal..

[27] Gómez-Rubio V., Rue H. (2018). Markov chain Monte Carlo with the Integrated Nested Laplace Approximation. Stat. Comput..

[28] Shrock E., Fujimura E., Kula T., Timms R.T., Lee I.H., Leng Y., Robinson M.L., Sie B.M., Li M.Z., Chen Y. (2020). Viral epitope profiling of COVID-19 patients reveals cross-reactivity and correlates of severity. Science.

[29] Kratzer B., Trapin D., Ettel P., Kormoczi U., Rottal A., Tuppy F., Feichter M., Gattinger P., Borochova K., Dorofeeva Y. (2020). Immunological imprint of COVID-19 on human peripheral blood leukocyte populations. Allergy.

[30] Barrett J.R., Belij-Rammerstorfer S., Dold C., Ewer K.J., Folegatti P.M., Gilbride C., Halkerston R., Hill J., Jenkin D., Stockdale L. (2021). Phase 1/2 trial of SARS-CoV-2 vaccine ChAdOx1 nCoV-19 with a booster dose induces multifunctional antibody responses. Nat. Med..

[31] Helman S.K., Mummah R.O., Gostic K.M., Buhnerkempe M.G., Prager K.C., Lloyd-Smith J.O. (2020). Estimating prevalence and test accuracy in disease ecology: How Bayesian latent class analysis can boost or bias imperfect test results. Ecol. Evol..

[32] Wolfe N.D., Dunavan C.P., Diamond J. (2007). Origins of major human infectious diseases. Nature.

[33] Borrega R., Nelson D.K.S., Koval A.P., Bond N.G., Heinrich M.L., Rowland M.M., Lathigra R., Bush D.J., Aimukanova I., Phinney W.N. (2021). Cross-Reactive Antibodies to SARS-CoV-2 and MERS-CoV in Pre-COVID-19 Blood Samples from Sierra Leoneans. Viruses.

[34] Mok C.K.P., Zhu A., Zhao J., Lau E.H.Y., Wang J., Chen Z., Zhuang Z., Wang Y., Alshukairi A.N., Baharoon S.A. (2021). T-cell responses to MERS coronavirus infection in people with occupational exposure to dromedary camels in Nigeria: An observational cohort study. Lancet Infect. Dis..

[35] El-Kafrawy S.A., Corman V.M., Tolah A.M., Al Masaudi S.B., Hassan A.M., Muller M.A., Bleicker T., Harakeh S.M., Alzahrani A.A., Alsaaidi G.A. (2019). Enzootic patterns of Middle East respiratory syndrome coronavirus in imported African and local Arabian dromedary camels: A prospective genomic study. Lancet Planet. Health.

[36] Che X.Y., Qiu L.W., Liao Z.Y., Wang Y.D., Wen K., Pan Y.X., Hao W., Mei Y.B., Cheng V.C., Yuen K.Y. (2005). Antigenic cross-reactivity between severe acute respiratory syndrome-associated coronavirus and human coronaviruses 229E and OC43. J. Infect. Dis..

[37] Chan K.H., Cheng V.C., Woo P.C., Lau S.K., Poon L.L., Guan Y., Seto W.H., Yuen K.Y., Peiris J.S. (2005). Serological responses in patients with severe acute respiratory syndrome coronavirus infection and cross-reactivity with human coronaviruses 229E, OC43, and NL63. Clin. Diagn. Lab. Immunol..

[38] Chan K.H., Chan J.F., Tse H., Chen H., Lau C.C., Cai J.P., Tsang A.K., Xiao X., To K.K., Lau S.K. (2013). Cross-reactive antibodies in convalescent SARS patients’ sera against the emerging novel human coronavirus EMC (2012) by both immunofluorescent and neutralizing antibody tests. J. Infect..

[39] Zhu Y., Yu D., Han Y., Yan H., Chong H., Ren L., Wang J., Li T., He Y. (2020). Cross-reactive neutralization of SARS-CoV-2 by serum antibodies from recovered SARS patients and immunized animals. Sci. Adv..

[40] Nkuba Ndaye A., Hoxha A., Madinga J., Marien J., Peeters M., Leendertz F.H., Ahuka Mundeke S., Arien K.K., Muyembe Tanfumu J.J., Mbala Kingebeni P. (2021). Challenges in interpreting SARS-CoV-2 serological results in African countries. Lancet Glob. Health.

[41] Steinhardt L.C., Ige F., Iriemenam N.C., Greby S.M., Hamada Y., Uwandu M., Aniedobe M., Stafford K.A., Abimiku A., Mba N. (2021). Cross-Reactivity of Two SARS-CoV-2 Serological Assays in a Setting Where Malaria Is Endemic. J. Clin. Microbiol..

[42] Lapidus S., Liu F., Casanovas-Massana A., Dai Y., Huck J.D., Lucas C., Klein J., Filler R.B., Strine M.S., Sy M. (2022). Plasmodium infection is associated with cross-reactive antibodies to carbohydrate epitopes on the SARS-CoV-2 Spike protein. Sci. Rep..

