# Supplementary material for: Identification of Serum Antibodies Cross-Reactive with Pathogenic Betacoronavirus Amongst Rural Communities Within the Forested Region of the Republic of Guinea
Source: Viruses. 2026 Sep 8;18(9):989. doi: 10.3390/v18090989 (PMC13612045; doi:10.3390/v18090989)

Supplemental Data

Supplementary Figure S1: Serological response to seasonal CoV glycoproteins

MSD analysis was performed on 103 UK negative controls (Negative), 517 rural forested region samples (Macenta), 66 and 94 samples from the urban areas of Coyah or Gueckedou and 51 UK COVID-19 convalescent samples (COVID). The responses to various spike glycoproteins are displayed as follows A) hCoV-229E, B) hCoV-NL63, C) hCoV-HKU1 D) hCoV-OC43. Data are shown as a calculated concentration using a known reference serum and a 4PL fit curve.

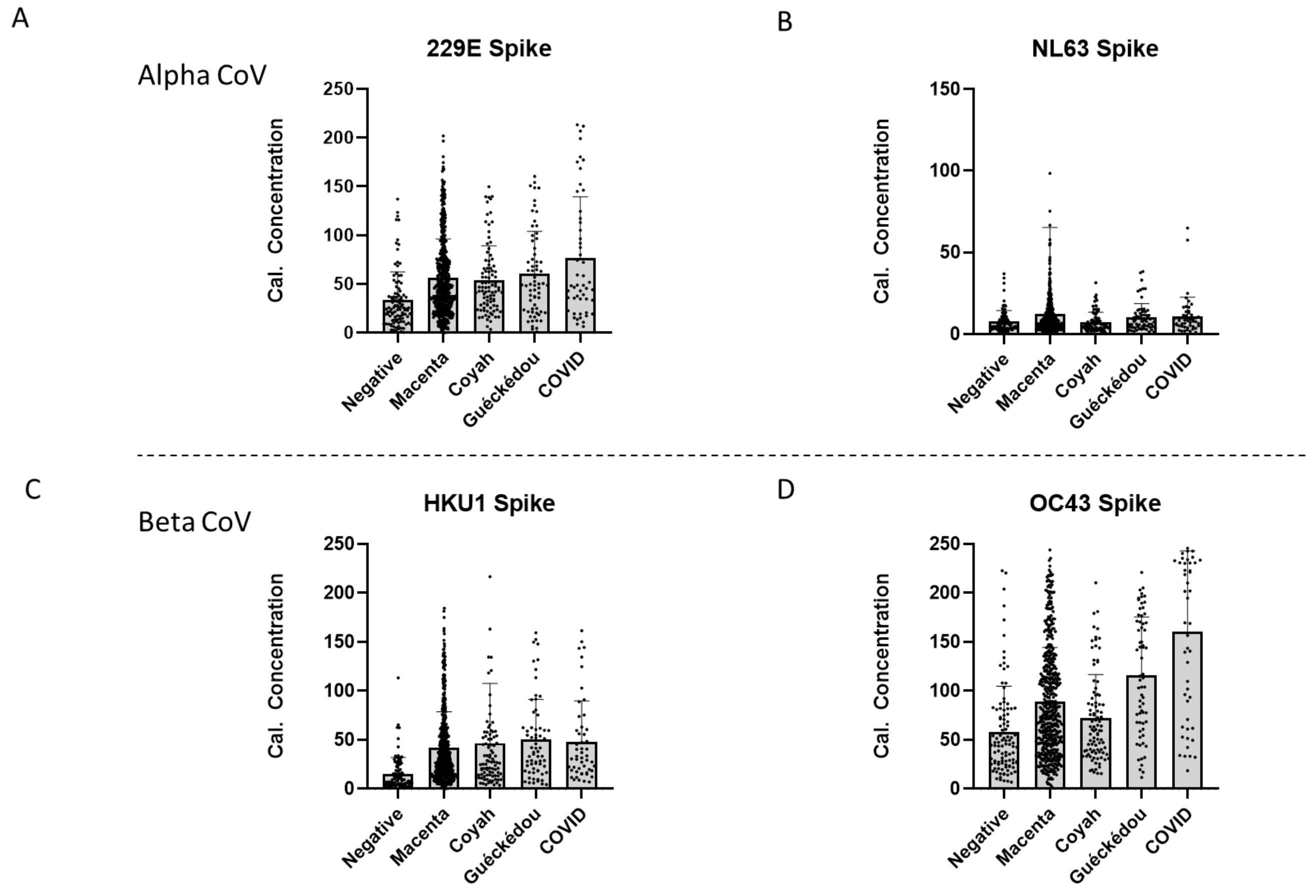

Supplementary Table S1: Percentage of samples above MSD threshold

This table records the percentage of each cohort that falls above our in house cut off values for the multiplexed MSD assay.

	Negative	Macenta	Gueckadou	Coyah	COVID
MERS Spike	0.97%	5.20%	6.06%	2.13%	29.41%
SARS-CoV-1 Spike	0.97%	4.43%	0.00%	1.06%	76.47%
SARS-CoV-2 Spike	0.97%	2.50%	1.52%	0.00%	92.16%
SARS-CoV-2 RBD	2.91%	3.08%	1.52%	1.06%	86.27%
SARS-CoV-2 NP	1.94%	21.19%	15.15%	9.57%	90.20%

Supplementary Figure S2: Correlation matrix for all serological analysis performed

Correlation between each serological parameter as measured by either ELISA or MSD. scale represents the R2 value between each parameter, with darker blue squares having a stronger positive correlation.

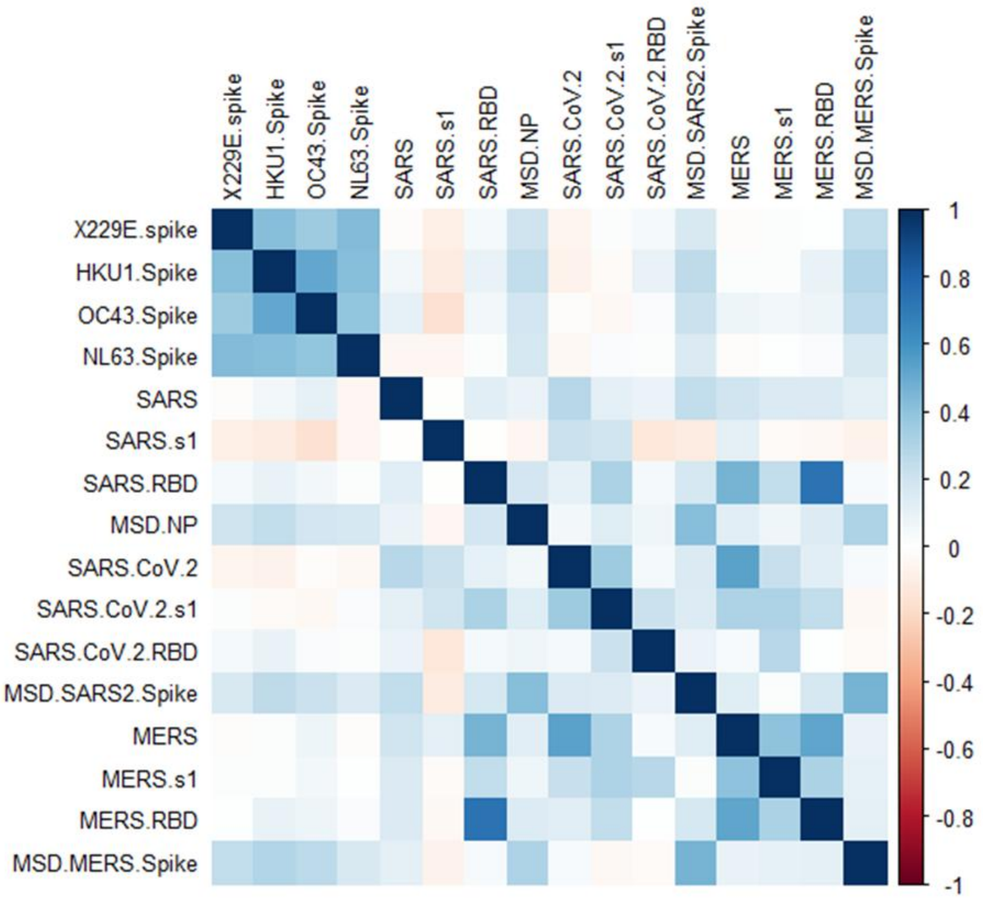

Supplementary Figure S3: In house ELISA QC data

Our in-house ELISA QC data. QC material used for these assays are CR3022, a Mab known to bind SARS-CoV, Convalescent SARS-CoV-2 human serum (N=1) and MERS-S1 a known MERS convalescent serum sample.

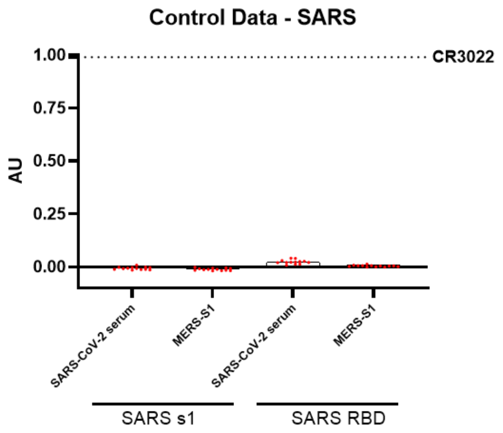

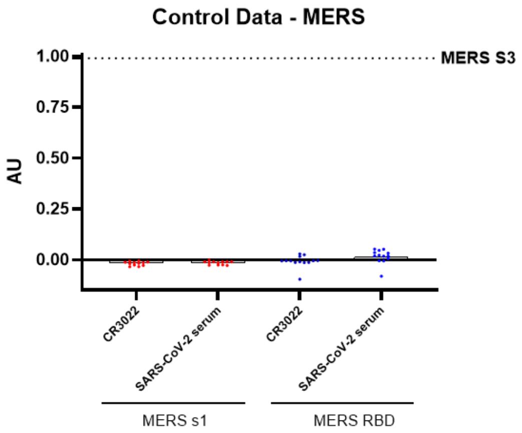

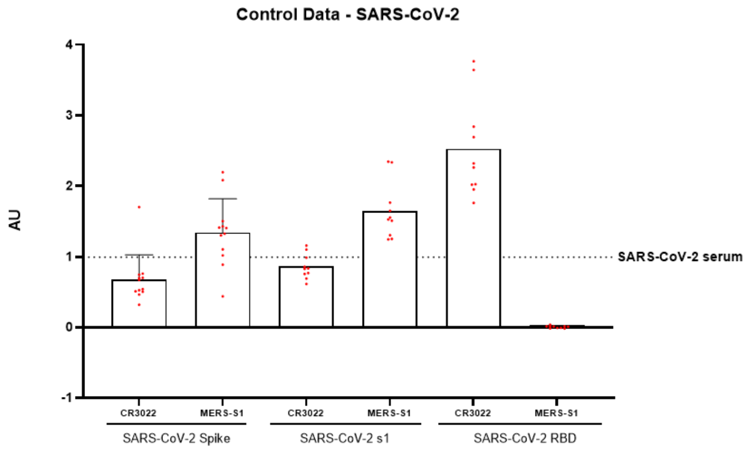

Supplementary Table S2: In house ELISA data percentage above cut off

This table records the percentage of each cohort that falls above the cut off value for our in-house ELISA assay.

	Negative	Coyah	Gueckadou	Macenta	COVID	Cut off
**Number**	111	97	69	515	20	5SD
**SARS Spike**	0.9%	1.0%	2.9%	4.1%	20.0%	0.014
**SARS s1**	0.0%	0.0%	2.9%	1.2%	15.0%	0.018
**SARS RBD**	1.8%	1.0%	0.0%	6.2%	5.0%	0.088
**SARS2 Spike**	0.9%	1.0%	1.4%	1.2%	60.0%	0.154
**SARS2 s1**	0.0%	1.0%	1.4%	4.3%	75.0%	0.224
**SARS2 RBD**	0.9%	0.0%	0.0%	1.0%	70.0%	0.229
**MERS spike**	0.9%	1.0%	8.7%	8.0%	0.0%	0.051
**MERS s1**	0.0%	2.1%	2.9%	2.5%	0.0%	0.024
**MERS RBD**	0.9%	1.0%	1.4%	20.4%	0.0%	0.112

Supplementary Figure S4: C3 complement deposition for MERS antigens

Comparison between IgG deposition and complement component C3 deposition on carboxyl beads coated with either MERS spike antigen (A) or MERS RBD antigen (B). Green bars indicate MERS sample S3 with is a single convalescent MERS patient.

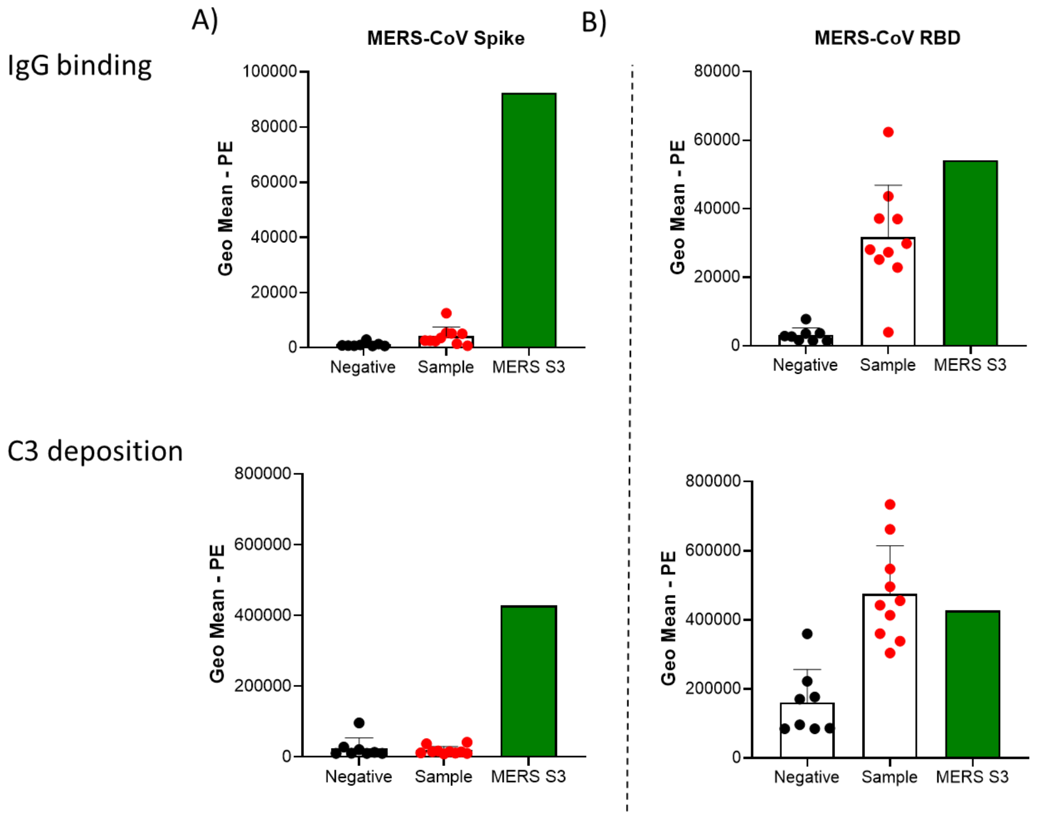

Supplementary Figure S5: Identification of exposed individuals to various antigens.

Bayesian latent class analysis (BLCA) to identify subpopulations of exposed individuals within samples collected from Guinea. BLCA is a method of model-based clustering used to identify homogenous sub-groups within populations and is widely applied to estimate infection prevalence from diagnostic tests without gold standards.

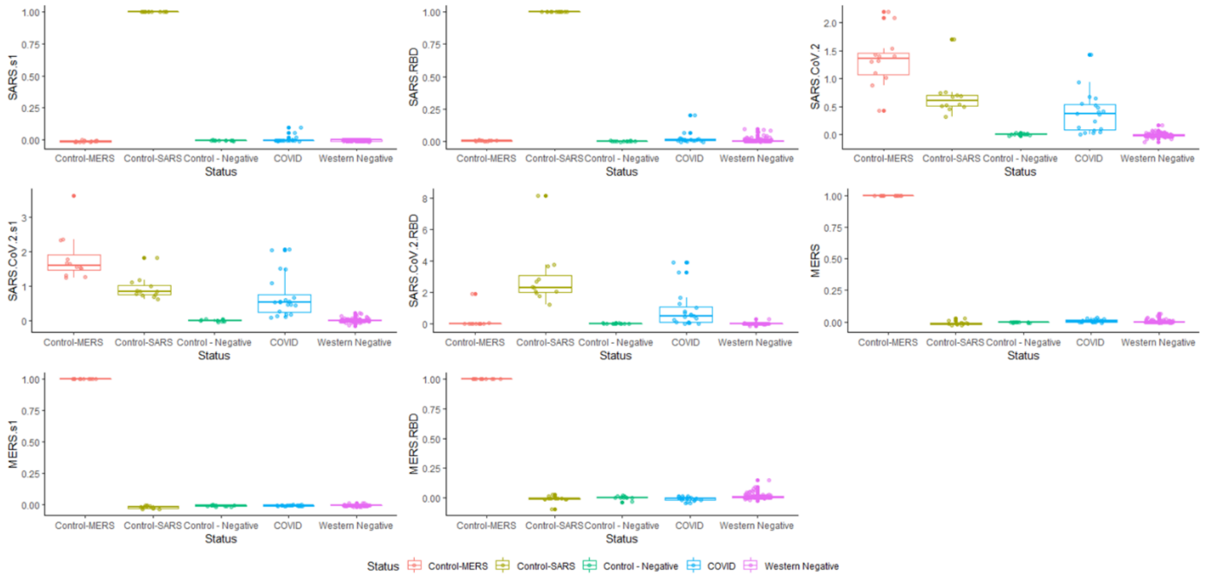

Supplementary Table S3: Diagnostic item probabilities conditional on class membership for four subgroups of viral exposures

Four distinct groups were identified using BCLA analysis, these are; those that had a low response to all CoV antigens (group 1), Those that responded only to seasonal CoV (group 2), those that had an elevated response to SARS-CoV-1 and MERS-CoV antigens (group 3) and those that had an elevated response to SARS-CoV-2 antigens (group 4). BLCA, conditional probabilities are equivalent to the sensitivity of the diagnostic test to predict membership within a specific subgroup

Antigen/ Assay	Group 1	Group 2	Group 3	Group 4
	Low responses to all CoV	Responses to seasonal CoV only	Elevated responses to SARS/ MERS antigens	Elevated responses to SARS-CoV-2 antigens
SARS s1/s2 ELISA	0.054	0.012	0.082	0.493
SARS s1 ELISA	0.018	0.046	0.027	0.129
SARS RBD ELISA	0.019	0.057	0.791	0.122
NP MSD	0.205	0.417	0.342	0.399
SARS-CoV-2 s1/s2 ELISA	0.007	0.041	0.028	0.764
SARS-CoV-2 s1 ELISA	0.002	0.026	0.025	0.433
SARS-CoV-2 RBD ELISA	0.014	0.021	0.032	0.090
SARS-CoV-2 spike MSD	0.036	0.059	0.066	0.368
MERS s1/s2 ELISA	0.045	0.072	0.672	0.682
MERS s1 ELISA	0.011	0.041	0.035	0.104
MERS RBD ELISA	0.146	0.143	0.970	0.318
MERS spike MSD	0.029	0.035	0.027	0.105
X229E spike MSD	0.150	0.471	0.159	0.129
HKU1 spike MSD	0.162	0.823	0.156	0.211
OC43 spike MSD	0.211	0.906	0.301	0.275
NL63 spike MSD	0.022	0.288	0.057	0.070

Supplementary Figure S6: BCLA analysis, spatial correlation to other antigens

Mean posterior estimate of probability and variance of individuals belonging to the subgroup with elevated responses to SARS S1/S2, MERS S1/S2, SARS RBD and MERS RBD ELISA as well as seasonal CoV MSD data.

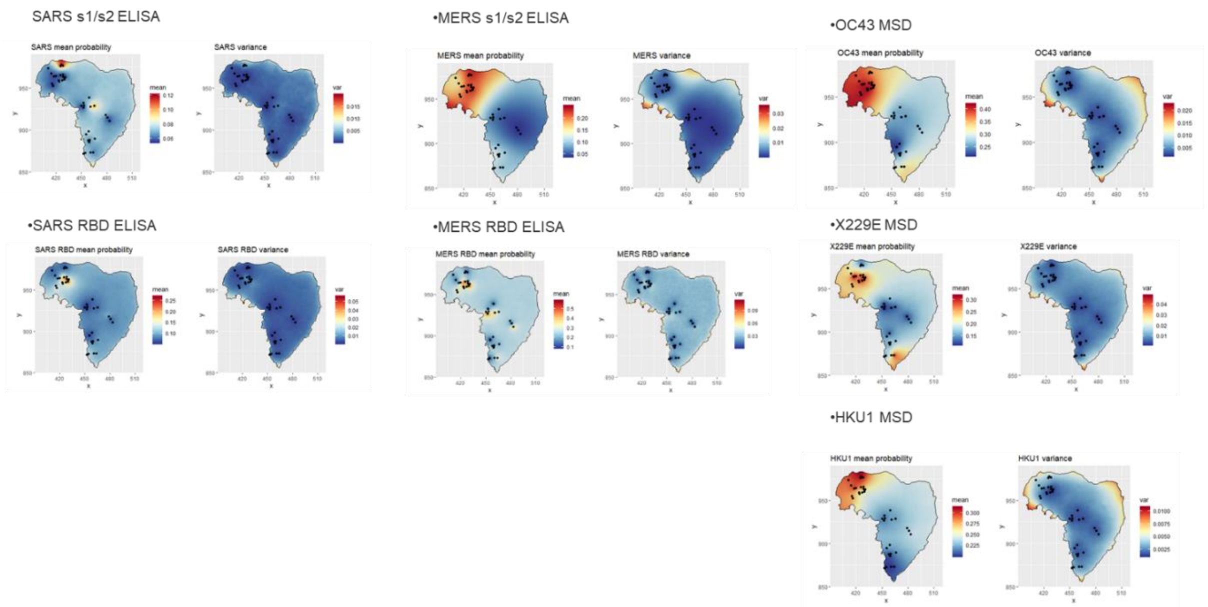

Supplementary Figure S7: ACE2i and Live virus neutralisation results

A

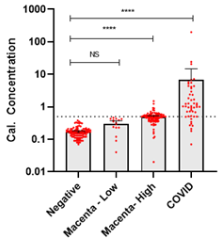

B

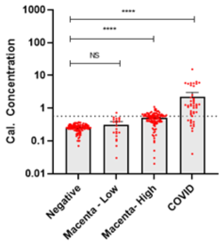

C

Virus	NT50		
	21/338	SL/MG	19/180
SARS-CoV-2 (VIC)	5867	10147	<20
MERS-CoV (Jordan)	<20	<20	120

Functional activity of cross-reactive samples. **A & B)** MSD ACE2 inhibition assay results. **C)** Live virus neutralisation assay positive control results.

Supplementary Figure S8: PepperPrint data for other CoV

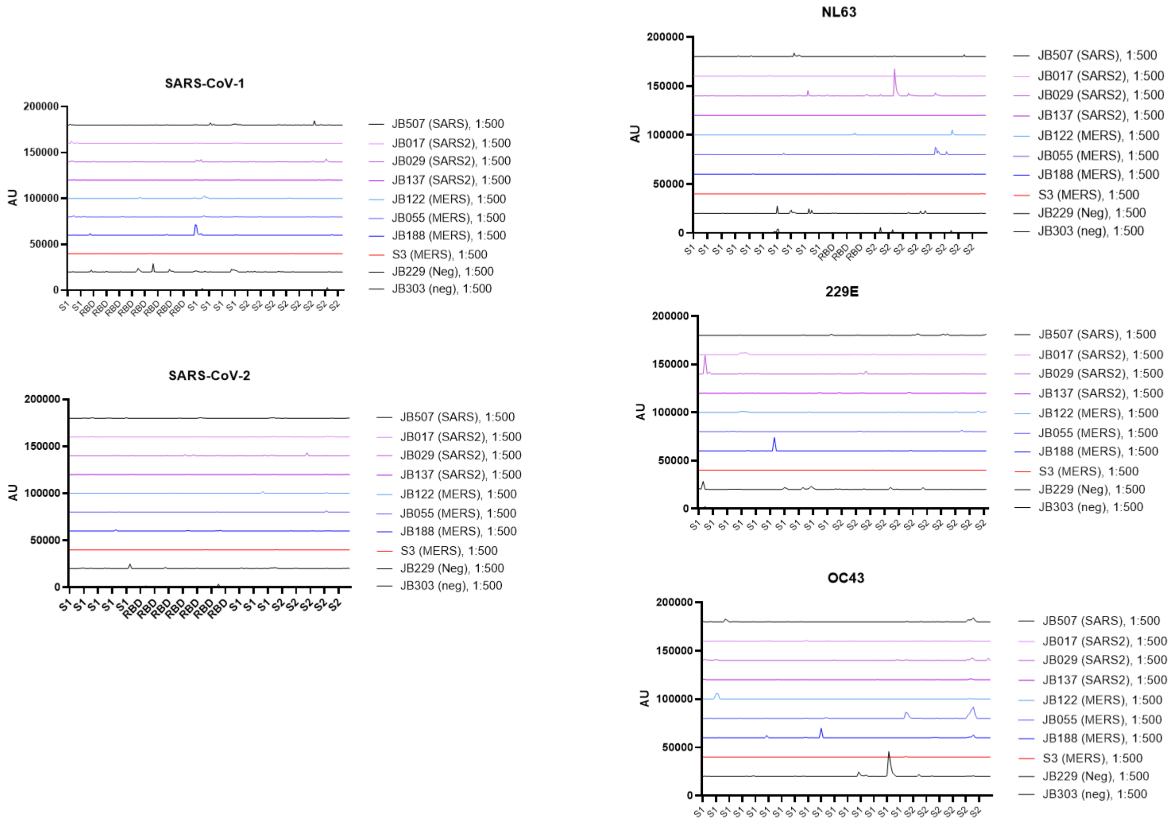

Supplementary Figure S9: Homology of peptide for other CoV

Homology between the MERS CoV sequence LRNCTFMYTYNITED which serum sample JB122 was found to bind and other SARS and seasonal CoV.

```
sp|Q6Q1S2|SPIKE_CVHNL            NCNG--YQHNS-VVDVM----RYNLNFSANS---LDNLKSGV--------IVFKTLQYDV   324
sp|P15423|SPIKE_CVH22            DCKG--FSSDV-LSDVI----RYNLNFEE-------NLRRGT--------ILFKTSYGVV   141
sp|P36334|SPIKE_CVHOC            SC-------------------LYKRNFTYDVN--AD-----YLY------FHFYQEGGTF   223
tr|U3NAI2|U3NAI2_CVHK1           LC-------------------LFKKNFTYNVS--TD-----FLY------FHFYQERGTF   209
tr|A0A140AYZ5|A0A140AYZ5_MERS    DCSDGNYNRNASLNSFKEYFNLRNCTFMYTYNITEDEILEWFGITQTAQG-VHLFSSRYV   271
sp|P0DTC2|SPIKE_SARS2            --------KSWMESEFRVYSSANNCTFEYVSQPFLMDLEGKQGNFKNLREFVFKNIDGYF   201
sp|P59594|SPIKE_SARS             ------------QTHTMIFDNAFNCTFEYISDAFSLDVSEKSGNFKHLREFVFKNKDGFL   194
```

JB122 - LRNCTFMYTYNITED

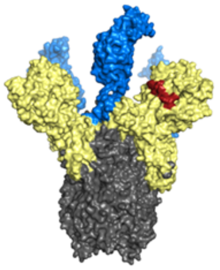

Supplement: Supplementary file 1 [file viruses-18-00989-s001.zip › viruses-4451873-supplementary.pdf]
